# Malaria Among Members of the U.S. Armed Forces, 2023

**Published:** 2024-05-20

**Authors:** Armed Forces Health Surveillance Division Defense Health Agency, US

## Abstract

*MSMR* publishes annual updates on the incidence of malaria among U.S. service members. Malaria infection remains a potential health threat to U.S. service members located in or near endemic areas due to duty assignment, participation in contingency operations, or personal travel. In 2023, a total of 39 active and reserve component service members were diagnosed with or reported to have malaria, an 8.3% increase from the 36 cases identified in 2022. Over half of the malaria cases in 2023 were caused by *Plasmodium falciparum* (53.8%; n=21) followed by unspecified types of malaria (35.9%; n=14) and *P vivax* and other *Plasmodia* (5.1%; n=2 each ). Malaria cases were diagnosed or reported from 22 different medical facilities: 18 in the U.S., 2 in Germany, 1 in Africa, 1 in South Korea. Of the 33 cases with known locations of diagnoses, 6 (18.2%) were reported from or diagnosed outside the U.S.

**What are the new findings?:**

Numbers of malaria cases began to increase after a low (n=20) in 2021, reaching 39 cases in 2023, mainly due to more from Africa as well as other or unspecified locations. Cases acquired in South Korea declined substantially in 2023.

**What is the impact on readiness and force health protection?:**

Malaria poses a health risk not only for service members deployed to endemic regions but those traveling to such areas for personal reasons. The finding that *P falciparum* malaria, which carries a high risk of serious sequelae, including death, was diagnosed in more than half of malaria cases in 2023 emphasizes the need for continued emphasis on effective preventive measures against this most dangerous malaria strain.

## 1 BACKGROUND

Malaria is a life-threatening disease spread to humans through the bites of *Anopheles* mosquitoes, found mostly in tropical countries.^1^ In 2022 the World Health Organization (WHO) reported that nearly half the world’s population was at risk of malaria, with an estimated 249 million malaria cases and 608,000 malaria deaths in 85 countries across different continents that present tremendous heterogeneity in the incidence of malaria deaths. Africa bears a disproportionately high share of the global malaria burden, with the vast majority (95%) of global malaria cases occurring there each year.^1,2,3^

Malaria is caused by 5 species of protozoan parasite of the genus *Plasmodium*: *P falciparum*, *P vivax*, *P malariae*, *P ovale*, or *P knowlesi*, of which *P falciparum* is most likely to cause severe infections and, if not promptly treated, may lead to death.^4^ While *P falciparum* is most prevalent in Africa, *P vivax* is the most widely geographically-distributed parasite species, with relatively high prevalence of infection in Southeast Asia, Western Pacific, and Eastern Mediterranean regions, as well as less densely populated areas of the Americas.^5^

Although malaria is not endemic in the United States, it is critical to monitor the incidence and trends of malaria among U.S. service members, due to potential health threats that may arise from being located in endemic areas due to duty assignments, participation in contingency operations, or personal travel.^6^ The *MSMR*’s focus on malaria reflects both historical lessons about this mosquito-borne disease and the continuing threat it poses to military operations and service members’ health.

The 2023 *MSMR* malaria update documented 12 individuals with exposures classified as deployment-related, of which 10 were classified as non-duty-related and 9 were considered acquired in Africa. Non-Hispanic Black service members accounted for 8 of those non-duty cases, and leisure travel to countries in Africa was documented in the reportable medical event (RME) records of 4 of these service members.^6^

Although malaria is a serious and potentially fatal disease, illness and death from malaria can be prevented by avoiding mosquito bites and proper use of malaria prophylaxis and standard preventive measures. The U.S. military has effective countermeasures against malaria, including chemoprophylactic drugs, permethrin-impregnated uniforms and bed nets, and DEET-containing insect repellents. Nevertheless, literature suggests that most malaria cases are associated with poor compliance with existing preventive measures.^7,8,9^

There is a need to identify gaps in existing approaches to more effectively combat malaria outbreaks and reflect changes in the incidence and trends of malaria infections among U.S. military personnel. This update describes the epidemiological patterns of malaria incidence among service members in the active and reserve components of the U.S. Armed Forces, using methods similar to those employed in previous analyses to explore factors for malaria prevention among this population.

## 2 METHODS

The surveillance period for this report was January 1, 2014 through December 31, 2023. The surveillance population included service members of the U.S. Army, Navy, Air Force, Marine Corps, Space Force, and Coast Guard.

The records of the Defense Medical Surveillance System (DMSS) were searched to identify qualifying evidence of a malaria diagnosis from RMEs, hospitalizations, outpatient encounters (in military and non-military facilities), and laboratory results from military facilities. Case definition criteria included 1) an RME record of confirmed malaria, 2) a hospitalization record with a primary diagnosis of malaria, 3) a hospitalization record with a non-primary diagnosis of malaria due to a specific Plasmodium species, 4) a hospitalization record with a non-primary diagnosis of malaria plus a diagnosis of anemia, thrombocytopenia, and related conditions, or malaria-complicating pregnancy in any diagnostic position, 5) a hospitalization record with a non-primary diagnosis of malaria plus diagnoses of signs or symptoms consistent with malaria in each diagnostic position preceding malaria, or 6) a positive malaria antigen test plus an outpatient record with a diagnosis of malaria in any diagnostic position within 30 days of the specimen collection date. The relevant International Classification of Diseases, 9th and 10th Revision (ICD-9/ICD-10) codes used to identify cases are shown in Table 1.

This analysis restricted each service member to 1 episode of malaria per 365-day period. When multiple records documented a single episode, the date of the earliest record was considered the date of clinical onset. Records within 30 days of the clinical onset date were reviewed for evidence of a *Plasmodium* species.

Presumed locations of malaria acquisition were estimated using a hierarchical algorithm: 1) cases diagnosed in a malaria-endemic country were considered acquired in that country, 2) RMEs that listed exposures to malaria-endemic locations were considered acquired in those locations, 3) RMEs that did not list exposures to malaria-endemic locations but were reported from installations in malaria-endemic locations were considered acquired in those locations, 4) cases diagnosed among service members during or within 30 days of deployment or assignment to a malaria-endemic country were considered acquired in that country, and 5) cases diagnosed among service members who had been deployed or assigned to a malaria-endemic country within 2 years before diagnosis were considered acquired in those respective countries. All remaining cases were considered to have acquired malaria in unknown locations.

## 3 RESULTS

**Figure 1:**
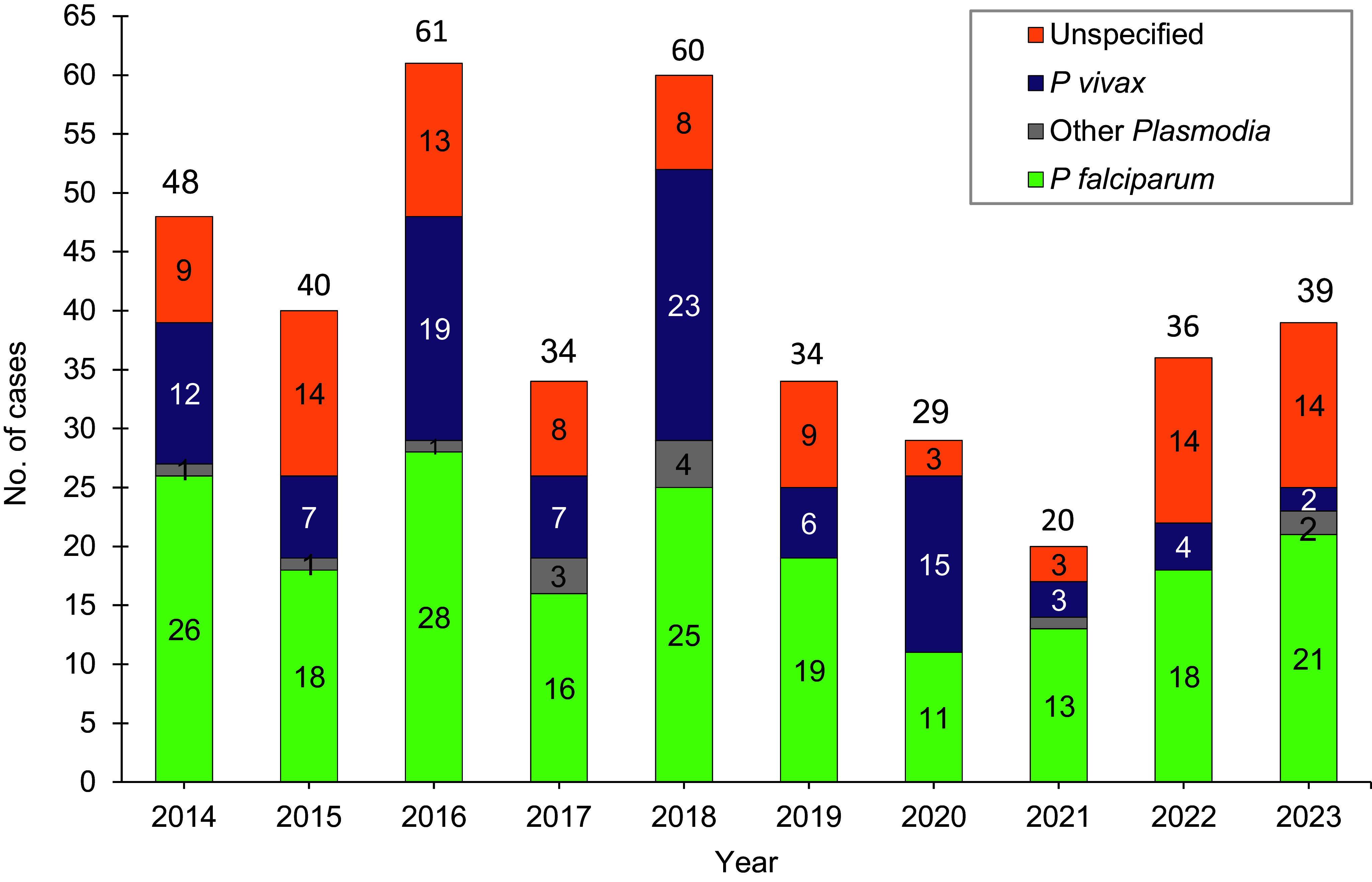
Numbers of Malaria Cases by Species and Calendar Year of Diagnosis or Report, Active and Reserve Components, U.S. Armed Forces, 2014-2023

In 2023, a total of 39 U.S. service members were diagnosed with or reported to have malaria (Table 2), resulting in a rate of 1.9 per 100,000 persons (data not shown). This total from 2023 represents an 8.3% increase from the 36 cases ascertained in 2022 (Figure 1). Twenty-one of the 39 cases (53.8%) in 2023 were identified from inpatient data reported as RMEs, while the remaining 18 cases were identified from inpatient data without associated RMEs. Six cases from 2023 were identified from laboratory data in combination with an outpatient record of malaria.

As in previous years, the majority of U.S. military members diagnosed in 2023 with malaria were men (92.3%), members of the active component (76.9%), and in the Army (69.2%). No cases from the Space Force nor Coast Guard were reported. Non-Hispanic Black service members and those under age 30 accounted for the most cases of malaria (79.5% and 35.9%, respectively) in 2023 (Table 2).

Of the 21 malaria cases reported as RMEs in 2023, all were male and 15 were in the Army. Of these 21 cases, 17 were categorized as non-duty-related travel, of which 16 were considered acquired in Africa. Non-Hispanic Black service members accounted for 13 of those nonduty cases.

**Figure 2:**
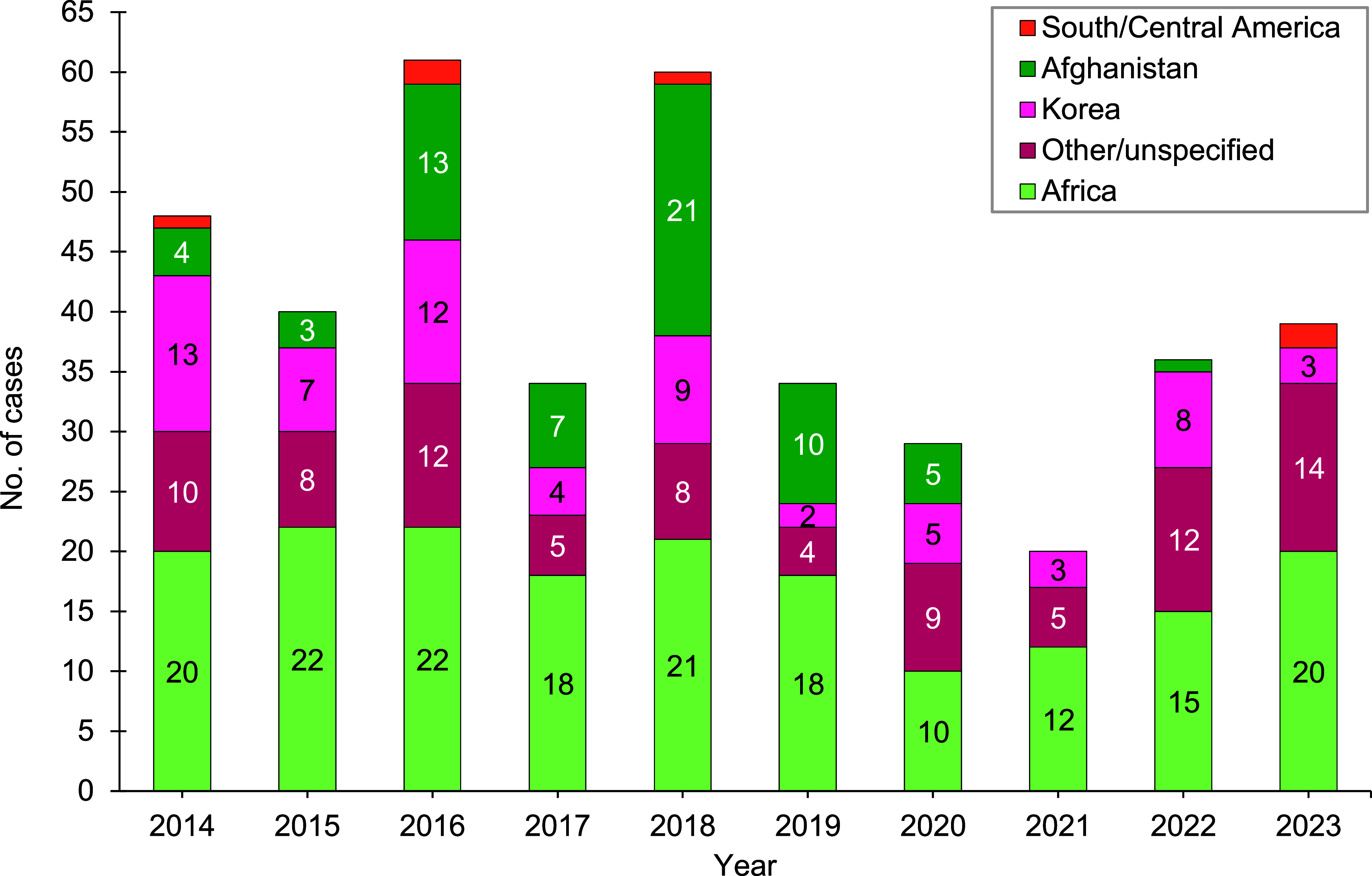
Numbers of Malaria Cases by Location of Acquisition, Active and Reserve Components, U.S. Armed Forces, 2014-2023

During the 2014-2023 surveillance period, malaria cases attributed to Africa accounted for the greatest number of cases (n=178; 44.4%), followed by other/unspecified locations (n=87; 21.7%), Korea (n=66; 16.5%), Afghanistan (n=64; 16%), and South and Central America (n=6; 1.5%) (Figure 2). The annual percentages of cases associated with Africa had the greatest variability, ranging from 34.5% in 2020 to 60.0% in 2021. From 2022 to 2023, the number of cases associated with Korea decreased the most, from 8 to 3. There was no case in Afghanistan in 2023.

Malaria cases were diagnosed or reported in 2023 from 22 different medical facilities in the U.S., Germany, Africa, and South Korea (Table 3).

Over half of the malaria cases in 2023 were caused by *P falciparum* (53.8%; n=21). Of the 18 cases not attributed to *P falciparum*, 2 (5.1%) cases each were caused by *P vivax* and other *Plasmodia*, while 14 were labeled as associated with other or unspecified types of malaria (35.9%) (Figure 1). This result reflects historical data over a 10-year surveillance period, where malaria cases caused by *P falciparum* have accounted for the largest number of cases (n=195; 48.6%) followed by *P vivax* (n=98; 24.4%), other/unspecified species (n=95; 23.7%), and other *Plasmodium* species (n=13; 3.2%). The annual percentages of cases attributed to *P vivax* during the surveillance period showed the greatest variability, ranging from 11.1% in 2022 to 51.7% in 2020.

**Figure 3:**
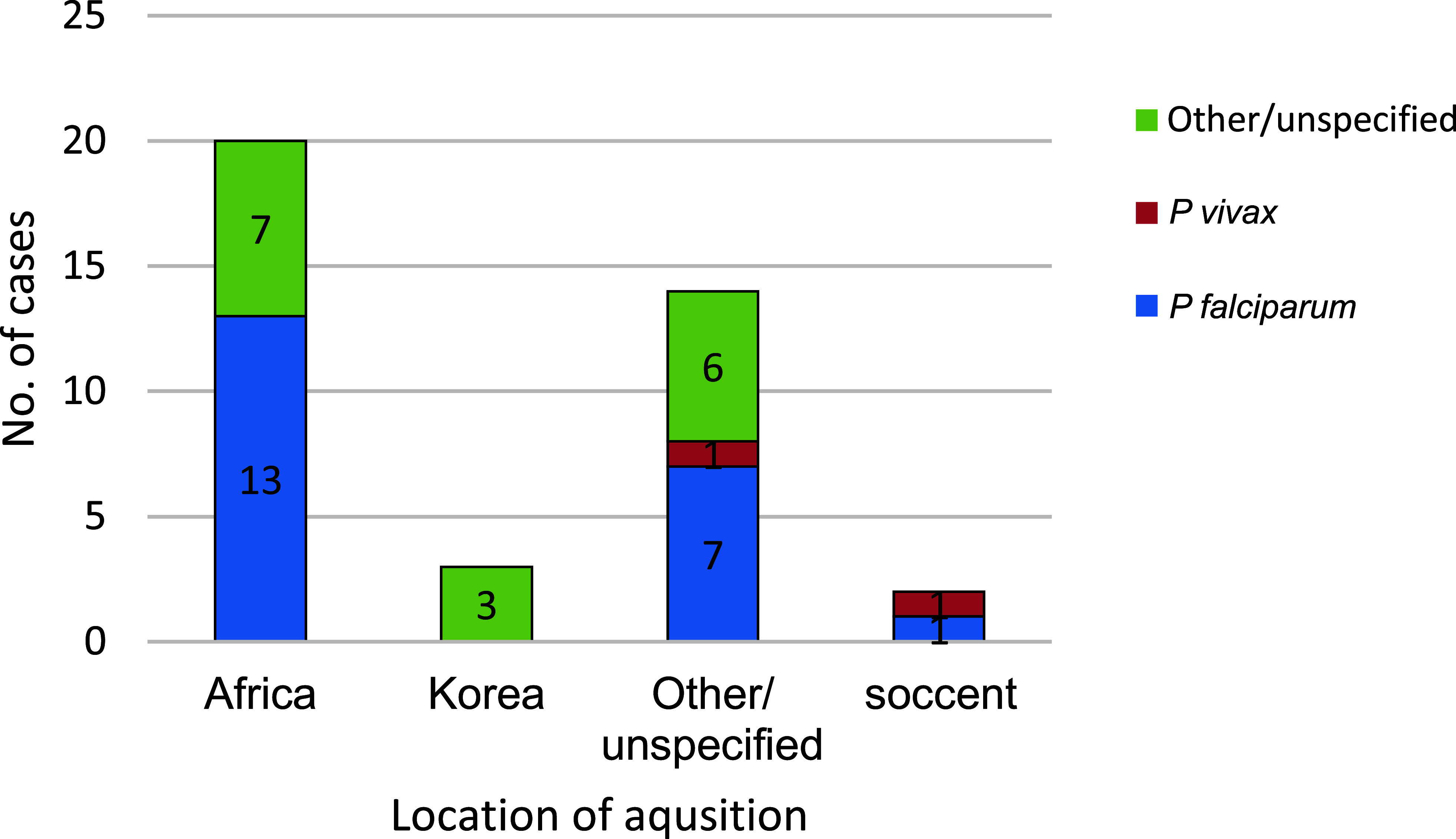
Numbers of Malaria Cases by Species Type and Location of Acquisition, Active and Reserve Components, U.S. Armed Forces, 2014-2023

Most cases acquired in Africa were caused by *P falciparum* (65.0%; 13/20) (Figure 3). Of the 20 malaria infections acquired in Africa in 2023, 3 each were linked to Djibouti and Sierra Leone; 2 were linked to Togo; 1 each were linked to Ghana, Côte d’Ivoire, and Nigeria; and 9 were associated with unknown African locations (data not shown).

**Figure 4:**
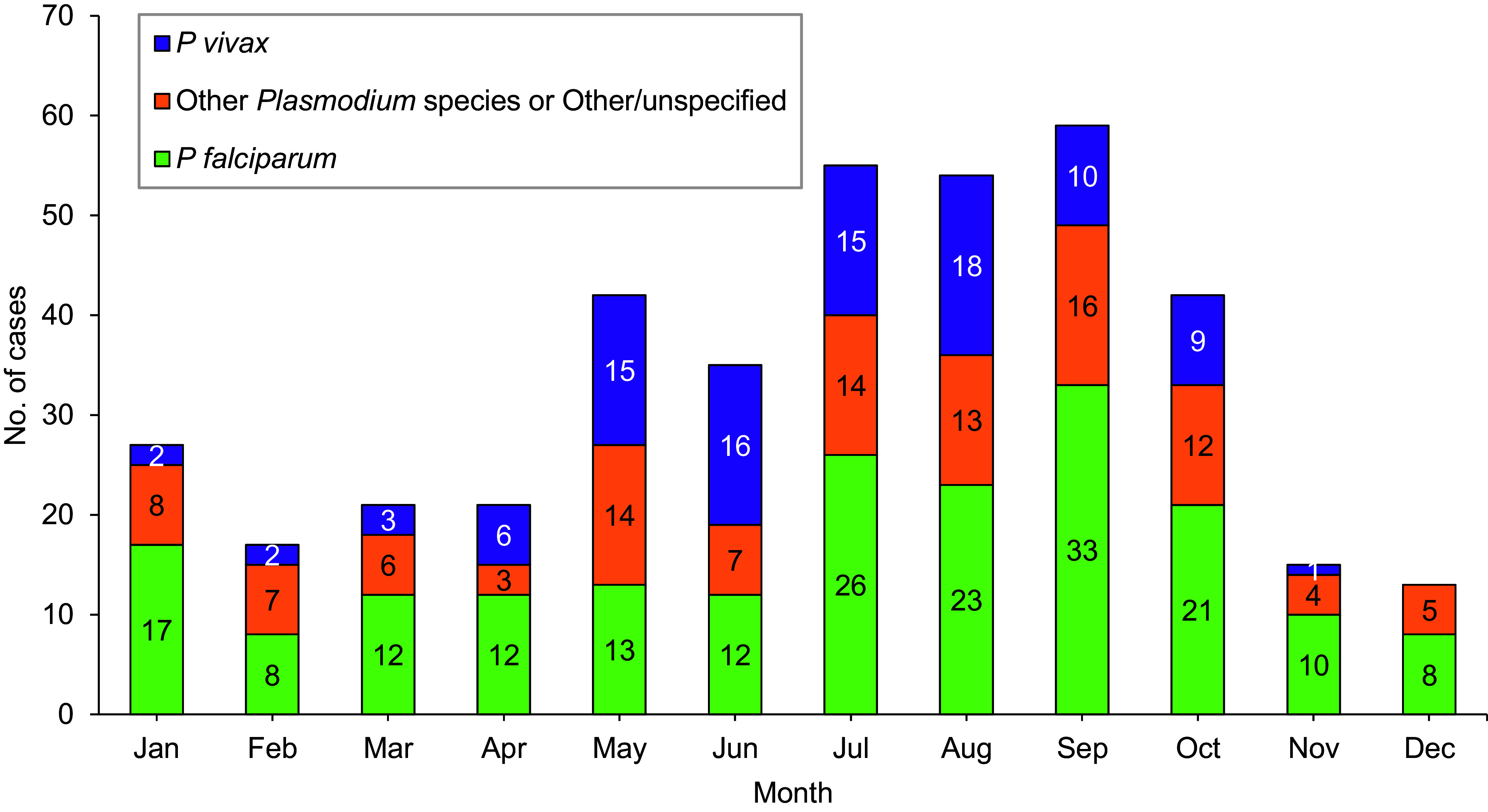
Cumulative Numbers of Malaria Cases by Species Type and Month of Clinical Presentation or Diagnosis, Active and Reserve Components, U.S. Armed Forces, 2014-2023

Between 2014 and 2023, most non-*P vivax* malaria cases (67.1%) were diagnosed or reported during the 6 months from the Northern Hemisphere middle of spring through the middle of autumn (May–October) (Figure 4). During the 10-year surveillance period, the proportions of non-*P vivax* malaria cases diagnosed or reported from May through October varied by region of acquisition: Korea (84.6%, 22/26), Afghanistan (84%, 21/25), Africa (67.8%, 116/171), and South and Central America (50.0%, 3/6) (data not shown).

## 4 DISCUSSION

Malaria remains an important infectious disease threat to U.S. service members deployed to tropical and subtropical regions due to operational constraints it imposes, lack of compliance with currently available preventive measures, and continuing emergence of drug-resistant malarial parasites.^10^ Although deployment-related exposures are targets for prevention, malaria poses a significant medical concern among service members who travel to malaria-endemic regions while on leave.^11,12^ In 2023 *P falciparum* was responsible for more than half of U.S. service member malaria cases, emphasizing the need for continued focus on prevention of the disease, given its potential severity and risk of death. Given that most RME-reported malaria infections occurred during non-duty-related activities and were acquired in Africa, it is important to effectively communicate malaria countermeasures to service members for both deployment and non-duty-related activities.

Several studies have reported low adherence with the recommended full course of prophylaxis and inadequate use of malaria chemoprophylaxis.^13,14,15^ Among the information regarding malaria patients and adherence to chemoprophylaxis, premature discontinuation of recommended chemoprophylaxis regimen upon completion of travel was given as a reason for non-adherence.^8^ Despite effective countermeasures against malaria and the success of mandatory chemoprophylaxis measures to prevent malaria, malaria infections will continue if service members do not adhere to the chemoprophylaxis necessary for its prevention. Completion of prophylaxis medication is necessary to prevent infection despite potential side effects. Efforts are needed to investigate side effects that may arise from these medications, for their effective mitigation, concurrent with efforts to identify factors that influence chemoprophylaxis adherence.

How risk management and prevention information is presented, and what specific or relevant information is provided, can have a greater impact on the knowledge, attitudes, and practices of deployed service members or travelers than basic information on what is prescribed.^8,16^ It is also necessary to consider the perceived risk of malaria infection during travel so service members do not mistakenly believe they have residual or innate immunity because they are visiting relatives, or that malaria treatment is easier than its prophylaxis.^14^ It is critical to explore more proactive approaches to assessing malaria risk and developing practical strategies according to identified risk factors to protect U.S. service members.^17^

Of particular concern is when foreign-born personnel travel on personal leave to their country of origin. A prior study demonstrated malaria rates 44 times greater for U.S. service members born in 7 western African countries than for those born in the U.S.^11^ Leisure travel to certain African countries, as reported on notifiable medical event records, may account, at least in part, for the disproportionately high malaria rates observed among non-Hispanic Black service members in this report. Among those service members visiting their birth countries in malaria-endemic regions, susceptibility due to loss of partial immunity from prior continuous exposure poses a substantial risk for infection and morbidity.^18^

Observations of seasonality in malaria diagnoses (Figure 4) indicate the need for more collaborative local and regional data collection efforts to quantify malaria seasonality and develop improved prevention strategies. Because *P falciparum* transmission is often seasonal, and a majority of non-*P vivax* malaria occurs during a 6-month period, from mid-spring to mid-autumn (May to October) in the Northern Hemisphere, accurate accounting of seasonality is important for informing efficient malaria control and treatment strategies.^19^ Surveillance for elimination purposes demands integration of related data, which allows for timely, targeted, and efficient resource deployment to prevent reintroduction of malaria to eliminated areas by mapping risk of receptivity and vulnerability.^20^

Limitations to this report should be considered when interpreting these findings. Malaria case documentation, especially for the reserve components as well as non-deployment-related exposures, is likely incomplete, leading to an underestimate of rates. Some cases treated in deployed or non-U.S. military medical facilities may not have been reported or otherwise ascertained at the time of this analysis. Malaria diagnoses recorded only in outpatient settings without confirmatory testing and not reported as notifiable events were not included. The geographic location where malaria was acquired was estimated from reported information; some cases had reported exposures in multiple malaria-endemic regions or areas, and others had no relevant exposure information. Personal travel or deployment within malaria-endemic countries was not accounted for unless specified in notifiable event reports. Limited information on species type in RME records reveals the need for greater attention on complete documentation of reportable conditions.

Military personnel frequently deploy to malaria-endemic regions, and most travel-related malaria cases occur due to non-compliance with preventive measures. Considering factors that can influence preventive measure compliance while promoting accurate awareness of malaria risk is critical. Positive perceptions of the necessity and efficacy of preventive measures, and appropriate reinforcement in relevant pre-travel advice, are important elements for continued prevention of malaria transmission to U.S. service members.

## Figures and Tables

**Table 1:** ICD-9 and ICD-10 Diagnosis Codes Used in Defining Malaria Cases from the Records for Inpatient Encounters (Hospitalizations)

	ICD-9	ICD-10
Malaria (*Plasmodium* species		
*P falciparum*	84.0	B50
*P vivax*	84.1	B51
*P malariae*	84.2	B52
*P ovale*	84.3	B53.0
Unspecified	84.4, 84.5, 84.6, 84.8, 84.9	B53.1, B53.8, B54
Anemia	280-285	D50-D53, D55-D64
Thrombocytopenia	287	D69
Malaria-complicating pregnancy	647.4	O98.6
Signs, symptoms, or other abnormalities consistent with malaria	276.2, 518.82, 584.9, 723.1, 724.2, 780.0, 780.01, 780.02, 780.03, 780.09, 780.1, 780.3, 780.31, 780.32, 780.33, 780.39, 780.6, 780.60, 780.61, 780.64, 780.65, 780.7, 780.71, 780.72, 780.79, 780.97, 782.4, 784.0, 786.05, 786.09, 786.2, 786.52, 786.59, 787.0, 787.01, 787.02, 787.03, 787.04, 789.2, 790.4	E87.2, J80, M54.2, M54.5, N17.9, R05, R06.0, R06.89, R07.1, R07.81, R07.82, R07.89, R11, R16.1, R17, R40, R41.0, R41.82, R44, R50, R51, G44.1, R53, R56, R68.0, R68.83, R74.0

Abbreviations: ICD-9, International Classification of Diseases, 9th Revision; ICD-10, International Classification of Diseases, 10th Revision; *P, plasmodium*

**Table 2:** Malaria Cases by *Plasmodium* Species and Selected Demographic Characteristics, U.S. Armed Forces, 2023

	*P vivax*	*P falciparum*	Other/Unspecified	Total	% Total	DMSS AC Reference Population^a^ (Oct. 2023)	
						n	%	
Total	2	21	16	39	100.0	2,102,128	100.0
Gender							
Male	2	20	14	36	92.3	1,699,479	80.8
Female	0	1	2	3	7.7	402,649	19.2
Age group, y							
<20	0	0	0	0	0.0	104,910	5.0
20-24	2	1	1	4	10.3	561,785	26.7
25-29	0	5	5	10	25.6	464,652	22.1
30-34	0	8	3	11	28.2	352,482	16.8
35-39	0	1	5	6	15.4	288,559	13.7
40-44	0	2	2	4	10.3	180,611	8.6
45-49	0	1	0	1	2.6	80,027	3.8
50+	0	3	0	3	7.7	69,102	3.3
Race and ethnicity							
White, non-Hispanic	1	1	3	5	12.8	1,159,050	55.1
Black, non-Hispanic	0	18	13	31	79.5	330,805	15.7
Other	1	2	0	3	7.7	235,945	11.2
Component							
Active	1	19	10	30	76.9	1,316,971	62.6
Reserve/Guard	1	2	6	9	23.1	785,157	37.4
Service							
Army	2	14	11	27	69.2	974,507	46.4
Navy	0	4	4	8	20.5	383,716	18.3
Air Force/Space Force	0	1	0	1	2.6	496,825	23.6
Marine Corps	0	2	1	3	7.7	201,964	9.6

Abbreviations: *P, plasmodium*; DMSS, Defense Medical Surveillance System; AC, all components; y, years

^a^Data Source: Defense Medical Surveillance System (DMSS) as of Feb. 14, 2024 prepared by the Defense Health Agency.

**Table 3:** Number of Malaria Cases by Geographic Location of Diagnosis or Report and Presumed Location of Acquisition, Active and Reserve Components, U.S. Armed Forces, 2023

Location Where Diagnosed or Reported	Korea	Afghanistan	Africa	South/Central America	Other/Unknown Location	Total	
	No.	No.	No.	No.	No.	No.	%
William Beaumont AMC, Fort Bliss, TX	3	0	2	0	0	5	12.8
Guthrie AHC, Fort Drum, NY	0	0	3	0	0	3	7.7
Womack AMC, Fort Liberty, NC	0	0	2	0	0	2	5.1
Carl R. Darnall AMC, Fort Cavazos, TX	0	0	2	0	0	2	5.1
NMC Portsmouth, VA	0	0	1	0	1	2	5.1
Landstuhl Regional Medical Center, Germany	0	0	0	0	2	2	5.1
Expeditionary Medical Center, Djibouti	0	0	2	0	0	2	5.1
Lyster AHC, Fort Novosel, AL	0	0	0	0	1	1	2.6
NMC San Diego, CA	0	0	1	0	0	1	2.6
Eisenhower AMC, GA	0	0	0	0	1	1	2.6
Martin ACH, Fort Moore, GA	0	0	0	1	0	1	2.6
Tripler AMC, HI	0	0	1	0	0	1	2.6
Walter Reed National NMC, MD	0	0	0	1	0	1	2.6
General Leonard Wood ACH, Fort Leonard Wood, MO	0	0	0	0	1	1	2.6
NMC Camp Lejeune, NC	0	0	1	0	0	1	2.6
Alexander T. Augusta MMC, Fort Belvoir, VA	0	0	0	0	1	1	2.6
Madigan AMC, Joint Base Lewis-McChord, WA	0	0	1	0	0	1	2.6
NHC Quantico, VA	0	0	1	0	0	1	2.6
Hohenfels AHC, Germany	0	0	1	0	0	1	2.6
DiRaimondo TMC, Fort Carson, CO	0	0	0	0	1	1	2.6
LaPointe AHC, Fort Campbell, KY	0	0	1	0	0	1	2.6
Henry L. Jenkins Medical Home, Camp Humphries, South Korea	0	0	1	0	0	1	2.6
Location not reported	0	0	0	0	6	6	15.4
Total	3	0	20	2	14	39	100

Abbreviations: No., number; AMC, Army Medical Center; AHC, Army Health Clinic; ACH, Army Community Hospital; NMC, Navy Medical Center; MMC, Military Medical Center; NHC, Naval Health Center; TMC, Troop Medical Center.

## References

[1] World Health Organization. (2023). World Malaria Report 2023..

[2] United Nations Foundation. United to Beat Malaria: About Malaria..

[3] Gao L, Shi Q, Liu Z, Li Z, Dong X (2023). Impact of the COVID-19 pandemic on malaria control in Africa: a preliminary analysis.. Trop Med Infect Dis..

[4] U.S. Centers for Disease Control and Prevention. Important Updates on Locally Acquired Malaria Cases Identified in Florida, Texas and Maryland..

[5] World Health Organization. (2022). World Malaria Report 2022..

[6] Armed Forces Health Surveillance Division. (2023). Malaria among members of the U.S. Armed Forces, 2013-2022.. MSMR..

[7] Beiter KJ, Wentlent ZJ, Hamouda AR, Thomas BN (2019). Nonconventional opponents: a review of malaria and leishmaniasis among United States Armed Forces.. PeerJ..

[8] Mace KE, Lucchi NW, Tan KR (2022). Malaria surveillance–United States, 2018.. MMWR Surveill Summ..

[9] Teneza-Mora N, Lumsden J, Villasante E (2015). A malaria vaccine for travelers and military personnel: requirements and top candidates.. Vaccine..

[10] United States Army Medical Materiel Agency. Malaria in the Military: Protecting our Warfighters..

[11] Wertheimer ER, Brundage JF, Fukuda MM (2011). High rates of malaria among US military members born in malaria-endemic countries, 2002–2010.. Emerg Infect Dis..

[12] Ashley DP, Fraser J, Yun H (2019). A comparison of pretravel health care, travel-related exposures, and illnesses among pediatric and adult U.S. military beneficiaries.. Am J Trop Med Hyg..

[13] Saunders DL, Garges E, Manning JE (2015). Safety, tolerability, and compliance with longterm antimalarial chemoprophylaxis in American soldiers in Afghanistan.. Am J Trop Med Hyg..

[14] Ahluwalia J, Brooks SK, Weinman J, Rubin GJ (2020). A systematic review of factors affecting adherence to malaria chemoprophylaxis amongst travellers from non-endemic countries.. Malar J..

[15] Collier RP, Lindholm DA, Lalani T (2021). 735. Malaria chemoprophylaxis adherence among U.S. active duty service members during deployment to endemic regions.. Open Forum Infect Dis..

[16] Hickey PW, Mitra I, Fraser J (2020). Deployment and Travel Medicine Knowledge, Attitudes, Practices, and Outcomes Study (KAPOS): malaria chemoprophylaxis prescription patterns in the Military Health System.. Am J Trop Med Hyg..

[17] White NJ (2011). Determinants of relapse periodicity in *Plasmodium vivax* malaria.. Malar J..

[18] Pousibet-Puerto J, Lozano-Serrano AB, Soriano-Pérez MJ (2021). Migration-associated malaria from Africa in southern Spain.. Parasit Vectors..

[19] Reiner RC, Geary M, Atkinson PM, Smith DL, Gething PW (2015). Seasonality of *Plasmodium falciparum* transmission: a systematic review.. Malar J..

[20] Hemingway J, Shretta R, Wells TN (2016). Tools and strategies for malaria control and elimination: what do we need to achieve a grand convergence in malaria?. PLoS Biology..

